# Emergence of extensively drug-resistant *Aeromonas hydrophila* complex isolated from wild *Mugil cephalus* (striped mullet) and Mediterranean seawater

**DOI:** 10.14202/vetworld.2022.55-64

**Published:** 2022-01-18

**Authors:** Hamza Mohamed Eid, Heba Sayed El-Mahallawy, Amany Mahmoud Shalaby, Hassnaa Mahmoud Elsheshtawy, Mera Mohamed Shetewy, Nada Hussein Eidaroos

**Affiliations:** 1 Department of Bacteriology, Immunology, and Mycology, Faculty of Veterinary Medicine, Suez Canal University, Ismailia 41522, Egypt; 2 Department of Animal Hygiene, Zoonoses, and Animal Behaviour and Management, Faculty of Veterinary Medicine, Suez Canal University, Ismailia 41522, Egypt; 3 Department of Food Hygiene, Animal Health Research Institute, Port Said Branch, Port Said, Egypt; 4 Department of Fish Diseases and Management, Faculty of Veterinary Medicine, Suez Canal University, Ismailia 41522, Egypt; 5 Veterinarian, Port Said, Egypt.

**Keywords:** *Aeromonas hydrophila* complex, antimicrobial resistance, *Mugil cephalus*, resistance genes, Seawater, virulence genes

## Abstract

**Background and Aim:**

Antibiotic resistance has been a progressively documented problem, resulting in treatment failure in humans and animals. This study aimed to investigate the antimicrobial susceptibility and virulence of extensively drug-resistant (XDR) *Aeromonas* spp. in wild *Mugil cephalus* and its surrounding seawater along the coastal road of Port Said, Egypt.

**Materials and Methods:**

Specimens were examined bacteriologically, confirmed biochemically, and tested for their sensitivity against 11 antimicrobial agents. Molecular confirmation of the obtained isolates by *16S rRNA* was performed, followed by the detection of antimicrobial resistance and virulence genes.

**Results:**

*Aeromonas* spp. was recovered from fish (44%) and water samples (36%). *A. hydrophila* was the most prevalent identified strain, followed by *Aeromonas sobria*, *Aeromonas caviae*, and *Aeromonas schubertii*. Moreover, 90% of the tested isolates were multidrug-resistant (MDR), while 26.67% were XDR. Tested isolates were resistant to b-lactams and sulfonamides (100%), oxytetracycline (90%), and streptomycin (62.22%) but completely susceptible to cefotaxime. XDR isolates successfully amplified resistance genes (*bla_TEM_ , sul1*, and *tetA*(*A*)) but not the (*aadA1*) gene, although there was phenotypic resistance to streptomycin on plates. All XDR isolates carry the cytotoxic enterotoxin gene (*act*), but *alt* gene was detected in only one isolate (12.5%).

**Conclusion:**

Data in this study provide a recent update and highlight the role of wild mullet and seawater as reservoirs for MDR and XDR *Aeromonas* spp. that may pose a risk to humans as food-borne infection or following direct contact.

## Introduction

*Aeromonas* species are facultative anaerobic Gram-negative bacteria that belong to the family *Aeromonadaceae* [1]. Although these bacteria are halotolerant waterborne pathogens and can tolerate water salinity of up to 3.0% NaCl, its presence in seawater is rare compared to that in river water [2,3]. *Aeromonas* species such as *Aeromonas caviae*, *Aeromonas veronii*, *Aeromonas hydrophila*, and *A. salmonicida* are responsible for most bacterial diseases in fish [4,5]. Motile *Aeromonas* septicemia (MAS) is one of the violent fish bacterial infections worldwide. It affects various species of fish and shellfish in both fresh and marine water, causing a serious problem for fish industry in Egypt and other countries [6]. Among *Aeromonas* species, *A. hydrophila* is the key causative agent of MAS in fish, causing mass mortality and massive economic losses [7-10]. These bacteria, known as opportunistic pathogens, can establish themselves only when fish are immunocompromised by variable stressors, such as overcrowding and concurrent disease [11].

Seawater has been identified as a source of various types of diseases worldwide. In 2003, cases of gastroenteritis and severe respiratory infection had been recognized in persons who swam or immersed themselves in seawater [12]. Motile *Aeromonas* are food-borne contaminant pathogens that have a microbial food safety concern due to their ability to grow at a wide range of temperatures, including refrigeration temperature, with the production of various virulence factors [13]. Infection may be acquired through drinking contaminated water, ingestion of raw or improperly cooked contaminated food, and close contact with infected animals [14,15]. In humans, the bacteria cause diseases ranging from mild to dysentery-like diarrhea to meningitis and septicemia [16]. Moreover, infection may occur extraintestinal, such as wound infections [17]. A recent study reported a multidrug-resistant (MDR) *A. hydrophila* to cause bilateral necrotizing fasciitis in an immunocompromised patient [18]. In general, *Aeromonas* spp., *Vibrio* spp., and *Shewanella* spp. had been identified to cause necrotizing fasciitis in patients after consumption of raw or undercooked seafood or following direct exposure to seawater along with coastal areas [19].

The enormous distribution of antibiotic resistance genes in nature among livestock, fish, vegetables, and plants makes it easily transmissible to humans through direct contact or ingestion of contaminated food [20]. This is facilitated by the high prevalence of gastrointestinal diseases in developing countries and routine use of antibiotics, resulting in the emergence of antibiotic resistance among enteric pathogens. In this way, horizontal gene transfer facilitates the spread of resistance even to other antibiotic classes, especially when the genes responsible for such resistance share the same transmissible element locations [21]. The ability of *Aeromonas* to cause disease is related to several complicated and multiple virulence factors [22], and *Aeromonas* virulence depends mainly on the secretion of exotoxins [23]. This includes a cytotoxic heat-labile enterotoxin (*act*) (known as aerolysin/hemolysin), cytotonic heat-labile enterotoxin (*alt*) (known as lipase, extracellular lipase, and phospholipase), and cytotonic heat-stable enterotoxin (*ast*) [24].

Antimicrobial resistance in marine water and its animals is always an issue of concern because they have no history of antimicrobial exposure [25]. Although numerous studies clarified the prevalence of *Aeromonas* spp. from *Mugil cephalus* in Egypt, there have been minimal data on mullets from the wild environments [26]. Port Said is a city in Egypt’s northeast region that extends approximately 30 km (19 miles) along the Mediterranean Sea’s coast, north of the Suez Canal. It is the most important source of fish to inhabitants of the city. *M. cephalus* is the widely consumed and preferable edible fish species in this area due to its high-quality flesh, large size, and superior growth. Moreover, fishing lovers in the city usually practice fishing along its coast and enjoy eating what they harvest.

This study aimed to determine the extent to which *Aeromonas* isolates from the wild environments are resistant to the most commonly used antibiotics among humans, animals, and fish in the study area and characterize the extensively drug-resistant (XDR) strains by detecting their antibiotic resistance genes and exploring enterotoxin production.

## Materials and Methods

### Ethical approval

Protocols for sample collection and laboratory testing for this study were reviewed and approved by the Scientific Research Committee and Bioethics Board of Suez Canal University, Faculty of Veterinary Medicine, Ismailia, Egypt.

### Study period and location

The samples were collected from July 2019 to September 2020. Samples were collected from Mediterranean seawater along the international coastal road of Port Said Governorate. Collection locations were the common places where local fishermen customarily collect fish in Port Said Governorate. The samples were processed at the Department of Food Hygiene Laboratory, Animal Health Research Institute, Port Said Branch, in Port Said Governorate, Egypt.

### Samples

A total of 100 apparently healthy *M. cephalus* were caught directly from seawater from different districts along the coast of Port Said Governorate. Moreover, 25 seawater samples (100 mL each) were also collected in sterile screw-capped colorless glass bottles from the same locations where fish had been caught. Fish and water samples were transported in an icebox (4-8°C) to the Department of Food Hygiene laboratory, Animal Health Research Institute, Port Said Branch, in Port Said Governorate, under complete aseptic conditions for bacteriological examination as soon as possible. For each fish, swabs were collected from the surface, gills, and internal organs (liver, spleen, and kidney) for bacteriological examination.

### Isolation and identification of Aeromonas spp.

Fish swabs and water samples were enriched in alkaline peptone water (Oxoid, Hampshire, UK) that were incubated at 25-30°C for 24 h before being plated onto *Aeromonas* agar medium (Oxoid) that had been incubated aerobically at 37°C for 18-24 h [27]. Based on colony characteristic morphology, suspected typical colonies (convex translucent, pale green, 0.5-3 mm in diameter) were obtained and purified for further testing. A complete set of biochemical tests was performed for colonies that were negative in Gram staining, motile, and positive for oxidase. Aerokey II was used for complete identification and biotyping of isolates down to the species level, where each species has its unique pattern in biochemical reactions [28].

### Antimicrobial susceptibility testing

The disk diffusion method was used to test antibiotic susceptibility of isolates on Mueller-Hinton agar (Oxoid) using the most commonly used antimicrobials in human, animal, and aquaculture treatment in the study area. Briefly, pure colonies from pure 24 h old culture were inoculated into 5 mL of Mueller-Hinton broth (Oxoid) and incubated for 4-5 h until the turbidity was observed. Then, the bacterial suspension was adjusted to a density equivalent to 0.5 McFarland standard. Sterile cotton swabs were used to streak the surfaces of Mueller-Hinton agar plates with the bacterial suspension, and the plates were left for 30 min. Then, antibiotic disks were placed on the surface of the plates using an antibiotic dispenser and sterile forceps. The following antimicrobials were used: Ampicillin (AM) 10 μg, penicillin (P) 10 μg, streptomycin (S) 10 μg, sulfamethoxazole-trimethoprim (SXT) 25 μg, oxytetracycline (T) 30 μg, cefotaxime (CTX) 30 μg, norfloxacin (NOR) 10 μg, amikacin (AK) 30 μg, gentamicin (CN) 10 μg, nalidixic acid (NA) 30 μg, and chloramphenicol (C) 30 μg (Bioanalyse®, Turkey). Then, plates were incubated aerobically at 37°C for 24 h. The recommended diameter for the inhibition zone of the National Committee for Clinical and Laboratory Standards Institute was used to classify the isolates as resistant, intermediate, or sensitive [29].

### Detection of the type of antimicrobial resistance pattern for the isolates

MDR isolates were defined as those that were resistant to at least one agent from three or more antimicrobial classes; however, XDR was used to describe isolates that showed resistance to at least one antimicrobial agent in most antimicrobials but remained susceptible to two or fewer classes of antimicrobials. Pandrug resistance (PDR) was used to define bacterial isolates that were not susceptible to any antibiotic from all antimicrobial classes [30].

### Molecular confirmation and typing of *Aeromonas spp.* isolates

Using QIAamp DNA Mini Kit (Qiagen, Germany, GmbH), DNA was extracted from 200 μL of bacterial suspension based on kit instructions. Briefly, 200 μL lysis buffer was added to the bacterial suspension with 20 μL kit protease. After incubation at 56°C for 10 min, the suspension was passed through the silica membrane of the spin column. After several washing steps, DNA was finally eluted from the membrane by centrifugation using 100 μL elution buffer into a new clean 1.5 mL microcentrifuge tube that was kept at −20°C for further molecular investigation.

Primer sequences targeting *16S rRNA* (Metabion, Germany) were used for molecular confirmation of the *Aeromonas* genus. Primers (Metabion) targeting *bla_TEM_*, *sul1*, *tetA(A)*, and *aadA1* genes for the detection of antimicrobial resistance genes and *act*, *ast*, and *alt* genes for the detection of virulence (enterotoxin production) were used to investigate virulence of the strains according to the previously published polymerase chain reaction (PCR) protocols using EmeraldAmp GT PCR Mastermix (Takara, Japan) (Table-1) [31-35]. Positive isolates were identified by detecting specific amplification bands after the amplification product had been electrophoresed using 1.5% agarose gel.

**Table 1:** Oligonucleotide primer sequences, cycling conditions, and length of the amplified product from *Aeromonas* spp. isolates.

Genes	Primer sequences (5’- 3’)	PCR cycling condition	Length of amplified product	Reference
*16S rRNA*	CTACTTTTGCCGGCGAGCGG	Initial denaturation 94°C/5 min 35 cycles: Denaturation at 94°C/30 s	953 bp	[31]
	TGATTCCCGAAGGCACTCCC	Annealing at 50°C/40 s Extension at 72°C/50 s		
*act*	GGGTGACCACCACCAAGAACA	Initial denaturation 94°C/5 min 35 cycles: Denaturation at 94°C/30 s	232 bp	[32]
	AACTGACATCGGCCTTGAACTC	Annealing at 55°C/40 s Extension at 72°C/30 s		
*ast*	TCTCCATGCTTCCCTTCCACT	Initial denaturation 94°C/5 min 35 cycles: Denaturation at 94°C/30 s	331 bp	
	GTGTAGGGATTGAAGAAGCCG	Annealing at 55°C/40 s Extension at 72°C/40 s		
*alt*	TGACCCAGTCCTGGCACGGC	Initial denaturation 94°C/5 min 35 cycles: Denaturation at 94°C/30 s	442 bp	
	GGTGATCGATCACCACCAGC	Annealing at 55°C/40 s Extension at 72°C/45 s		
*bla*_TEM_	ATCAGCAATAAACCAGC	Initial denaturation 94°C/5 min 35 cycles: Denaturation at 94°C/30 s	516 bp	[33]
	CCCCGAAGAACGTTTTC	Annealing at 54°C/40 s Extension at 72°C/45 s		
*Sul1*	CGGCGTGGGCTACCTGAACG	Initial denaturation 94°C/5 min 35 cycles: Denaturation at 94°C/30 s	433 bp	[34]
	GCCGATCGCGTGAAGTTCCG	Annealing at 60°C/40 s Extension at 72°C/45 s		
*aadA1*	TATCAGAGGTAGTTGGCGTCAT	Initial denaturation 94°C/5 min 35 cycles: Denaturation at 94°C/30 s	484 bp	[35]
	GTTCCATAGCGTTAAGGTTTCAT	Annealing at 54°C/40 s Extension at 72°C/45 s		
*tetA (A)*	GGTTCACTCGAACGACGTCA	Initial denaturation 94°C/5 min 35 cycles: Denaturation at 94°C/30 s	576 bp	
	CTGTCCGACAAGTTGCATGA	Annealing at 50°C/40 s Extension at 72°C/45 s		

*act*=Cytotoxic heat-labile enterotoxin genes, *ast*=Cytotonic heat-stable enterotoxin genes, *alt*=Cytotonic heat-labile enterotoxin genes, *bla*_TEM_=b-lactamase ampicillin resistance gene, *sul1*=Sulfonamide resistance gene, *aadA1*=Streptomycin-resistant gene, and *tetA (A)*=Tetracycline resistance gene, PCR=Polymerase chain reaction

### Statistical analysis

Data were handled using Microsoft Excel 2013, and Statistical Package for the Social Sciences (SPSS) 20.0 (IBM-SPSS Inc., Chicago, IL, USA) was used in the data analysis. Variables were checked for normality using the Shapiro–Wilk test at 0.05 level. Accordingly, variables were significant (Shapiro–Wilk test<0.05), and data were non-parametric. Differences between groups were checked using Chi-square (χ^2^) test at p≤0.05.

## Results

### Occurrence of Aeromonas spp. in wild *M. cephalus* and seawater

**Figure-1:**
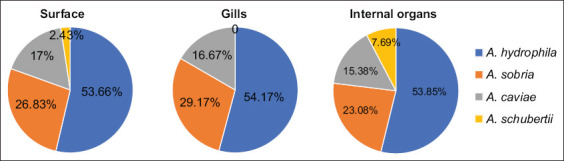
*Aeromonas* spp. recovered from different swabbing sites of *Mugil cephalus*.

Based on cultural characteristics and biochemical reactions, *Aeromonas* spp. were identified in 44 (44%) of 100 apparently healthy mullets and 9 (36%) of 25 seawater samples. A total of 97 isolates were obtained from mullet and seawater (78 and 19, respectively). The most frequently identified species from mullet was *A. hydrophila* (53.85%), followed by *Aeromonas sobria* (26.92%), *A. caviae* (16.67%), and *Aeromonas schubertii* (2.56%). The same sequence of occurrence was also observed in isolates from seawater; however, *A. schubertii* was not recovered (Table-2). Similarly, isolates obtained from samples of surface, gills, and internal organs of fish follow the same order of distribution for *Aeromonas* strains (Figure-1). Differences between the prevalence of these four *Aeromonas* spp. isolated from mullet and seawater and different swabbing sites of fish were highly statistically significant as revealed by χ^2^ test (p<0.001***). However, the specific presence of each *Aeromonas* spp. among different tissues and swabbing sites of the examined fish was not statistically significant as revealed by Kruskal–Wallis test (p>0.05).

**Table 2:** Identified *Aeromonas* spp. strains from *M. cephalus* and seawater.

Origin and no. of samples	Positive samples n (%)	Total isolates recovered	*A. hydrophila*	*A. sobria*	*A. caviae*	*A. schubertii*
			
			n (%)	n (%)	n (%)	n (%)
*M. cephalus* (n=100)	44 (44%)	78^a^	42 (53.85)	21 (26.92)	13 (16.67)	2 (2.56)
Surface		41	22 (53.66)	11 (26.83)	7 (17.07)	1 (2.43)
Gills		24	13 (54.17)	7 (29.17)	4 (16.67)	0 (0.0)
Internal organs		13	7 (53.85)	3 (23.08)	2 (15.38)	1 (7.69)
Seawater (n=25)	9 (36%)	19^a^	9 (47.37)	7 (36.84)	3 (15.79)	0 (0.0)

a Number of isolates exceeded the number of positive samples (44 from fish and nine from seawater) because some fish yielded more than 1 strain from different swabbing sites of its body. The same was also observed for water samples. *M. cephalus=Mugil cephalus, A. sobria=Aeromonas sobria, A. caviae=Aeromonas caviae, A. schubertii=Aeromonas schubertii, A. hydrophila=Aeromonas hydrophila*

### Antimicrobial sensitivity profile of bacterial isolates

**Figure-2:**
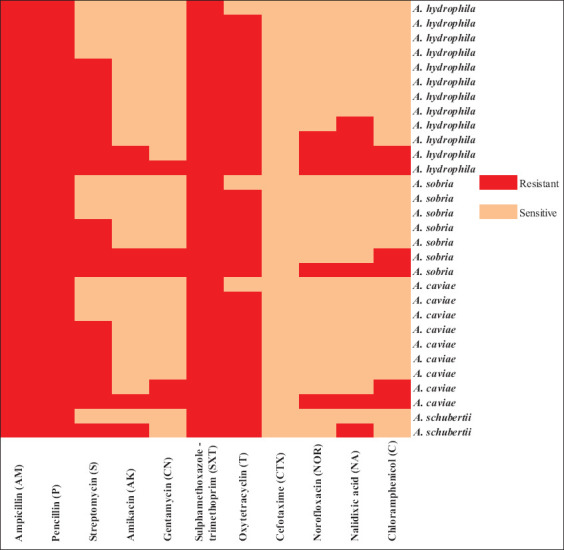
Heat map showing a summary of antibiogram for *Aeromonas* spp. isolates. Red blocks represent resistance, and light blocks represent sensitivity to the antimicrobial agent.

A total of 30 representative isolates (20 from *M. cephalus* and 10 from seawater) were tested against 11 types from seven different antimicrobials classes. Overall, the tested isolates exhibited complete resistance (100%) to AM, P, and SXT; extremely high resistance to T (90%); and considerable resistance to S (63.33%) (Table-3 and Figure-2). However, a low level of resistance (<25%) was observed for NOR, AK, CN, NA, and C. None of the isolates were found resistant to CTX. Overall, there was a statistically significant difference in the resistance profile exhibited by various *Aeromonas* spp. and the tested antimicrobial agents. This difference was highly significant with oxytetracycline, NOR, and CN (p<0.001***) and significant with AK, C, and NA (p=0.001** and p=0.003**) as revealed by the χ^2^ test.

**Table 3:** Antimicrobial resistance profile of *Aeromonas* spp. isolates.

Antimicrobial class	Antimicrobial agent	*A. hydrophila* (n=12)	*A. sobria* (n=7)	*A. caviae* (n=9)	*A. schubertii* (n=2)	No. of tested isolates (n=30)

		Resistant isolates n (%)				n (%)
b-lactams	AM^N/A^	12 (100)	7 (100)	9 (100)	2 (100)	30 (100)
	P^N/A^	12 (100)	7 (100)	9 (100)	2 (100)	30 (100)
Aminoglycosides	S	8 (66.67)	4 (57.14)	6 (66.67)	1 (50)	19 (63.33)
	AK**	2 (16.67)	2 (28.57)	1 (11.11)	1 (50)	6 (20)
	CN***	1 (8.33)	2 (28.57)	2 (22.22)	0 (0.0)	5 (16.67)
Sulfonamides	SXT^N/A^	12 (100)	7 (100)	9 (100)	2 (100)	30 (100)
Tetracyclines	T***	11 (91.67)	6 (85.71)	8 (88.89)	2 (100)	27 (90)
Cephalosporins	CTX^N/A^	0 (0.0)	0 (0.0)	0 (0.0)	0 (0.0)	0 (0.0)
Quinolones	NOR***	3 (25)	1 (14.29)	1 (11.11)	0 (0.0)	5 (16.67)
	NA**	4 (33.33)	1 (14.29)	1 (11.11)	1 (50)	7 (23.33)
Phenicols	C**	2 (16.67)	2 (28.57)	2 (22.22)	0 (0.0)	6 (20)

*** Highly statistically significant difference;

** statistically significant difference, ^N/A^ not applicable. *A. sobria*=*Aeromonas sobria*, *A. caviae*=*Aeromonas caviae*, *A. schubertii*= *Aeromonas schubertii*, *A. hydrophila=Aeromonas hydrophila,* AM=Ampicillin, P*=*Penicillin, S=Streptomycin, SXT=Sulfamethoxazole-trimethoprim, T=Oxytetracycline, CTX=Cefotaxime, NOR=Norfloxacin, AK=Amikacin, CN=Gentamicin, NA=Nalidixic acid, C=Chloramphenicol

### Antimicrobial resistance pattern of the tested isolates

Concerning the resistance pattern of the tested isolates (MDR, XDR, and PDR) in the present study, 27 of 30 isolates (90%) were MDR (11, *A. hydrophila*; 6, *A. sobria*; 8, *A. caviae*; and 2, *A. schubertii*), while eight of 30 isolates (26.67%) were XDR (4, *A. hydrophila*; 2, *A. sobria*; 1, *A. caviae*; and 1, *A. schubertii*), which were resistant to all examined antimicrobial agents, except one or two classes. None of the isolates were PDR.

### Molecular confirmation and typing of *Aeromonas* spp. isolates

Molecular verification of the recovered isolates using PCR targeting *16S rRNA* gene revealed *Aeromonas* spp. specific band at 953 bp on 1.5% agarose gel. XDR isolates (Figure-2) were subjected to molecular detection of virulence (enterotoxin production) genes (*alt, ast*, and *act*) and antibiotic resistance genes (*bla_TEM_, sul1, tetA(A)*, and *aadA1*) corresponding to the highest phenotypic resistance observed on plates to the first line of antimicrobial treatment in fish, animals, and humans in the study area. The results showed that all tested isolates (100%) possess the antibiotic resistance genes *bla_TEM_, sul1*, and *tetA(A)*; however, none had *aadA1* gene. The cytotoxic heat-labile *act* gene was the only virulence gene carried by all isolates; however, only one isolate of *A. sobria* from mullet had the cytotonic heat-labile *alt* enterotoxin gene (Table-4). None of the isolates showed the specific amplification band of *ast* gene.

**Table 4:** Occurrence of antimicrobial resistance genes and virulence genes (enterotoxin genes) among XDR *Aeromonas* spp. isolates.

Isolates of *Aeromonas* spp.	Source of the isolate	*bla_TEM_*	*sul1*	*tetA (A)*	*aadA1*	*alt*	*ast*	*act*
*A. hydrophila*	*M. cephalus*	+	+	+	−	−	−	+
*A. hydrophila*	Seawater	+	+	+	−	−	−	+
*A. hydrophila*	*M. cephalus*	+	+	+	−	−	−	+
*A. hydrophila*	Seawater	+	+	+	−	−	−	+
*A. sobria*	*M. cephalus*	+	+	+	−	+	−	+
*A. sobria*	Seawater	+	+	+	−	−	−	+
*A. schubertii*	*M. cephalus*	+	+	+	−	−	−	+
*A. caviae*	*M. cephalus*	+	+	+	−	−	−	+

*M. cephalus=Mugil cephalus, A. sobria=Aeromonas sobria, A. caviae=Aeromonas caviae, A. schubertii=Aeromonas schubertii,* *A. hydrophila*=*Aeromonas hydrophila*, XDR=Extensively drug resistant

## Discussion

This study reports the occurrence, virulence, and antibiogram of XDR motile *Aeromonas* in apparently healthy wild *M. cephalus* and seawater samples in Port Said Governorate and its probable hazard to consumers and fish handlers in the study area. Overall, the recovery rate of *Aeromonas* spp. varied from 44% in *M. cephalus* to 36% in seawater (Table-2). In Egypt, numerous studies have reported various occurrence rates of *Aeromonas* spp. from farmed, retail, and frozen fish (88% [36], 14.2% [37], 37% [38], and 30% [39]), but none of them reported its occurrence in wild fish or marine environment. In North Italy, *Aeromonas* spp. was isolated at a lower level (16%) from that in the coastal water of Mediterranean Sea (North Ionian Sea of Italy) [40].

Although *A. hydrophila* has gained the most attention, *A. caviae* and *A. veronii* are the known species most commonly isolated from clinical and environmental samples [41]. In the current report, *A. hydrophila* was the most prevalent species recovered from *M. cephalus* and seawater. However, this was not the usual pattern in farmed freshwater fish. In a recent study in Egypt, *A. sobri*a (44%), *A. cavia*e (28%), *A. hydrophil*a (20%), and *A. veroni*i (8%) were the most predominant species from mullet collected from different fish farms in Kafr El-Sheikh governorate [42]. However, another study indicated that *A. caviae* dominates in seawater fish (7.2%) [43]. In the current study, *A. schubertii* was the least identified *Aeromonas* spp. from *M. cephalus* (2/78, 2.56%). It seems that this species has a lower distribution in our area, as indicated in the previous studies [44,45]. A possible explanation for these differences is the geographical locations where several studies are conducted, variable environmental temperature, and chemical and physical characteristics of the water [46].

Data in the present study showed a higher occurrence of *Aeromonas* spp. in swabs from the surface of the fish and gills. Meanwhile, internal organs showed the lowest recovery rate (Table-2 and Figure-1). In contrast, *A. hydrophila* was more commonly recovered from internal organs than gills in an earlier report [47]. Regardless of the isolated *Aeromonas* species from different tissue sites of the fish, it is noteworthy that high recovery from the surface of the fish may represent a potential occupational hazard for fish handlers, aquaculture workers, housewives, and fishing fans through direct contact, especially if these strains are MDR.

Over the past decades, resistance has been developed to natural and synthetic antibiotics due to massive production and use of antibiotics, a matter that necessitates frequent investigation of resistant organisms in humans, animals, and the environment [48]. Findings in this study showed that 90% of the examined *Aeromonas* spp. isolates were MDR, and 26.67% were XDR. This agreed with the findings of Odeyemi and Ahmad [49] who found that 14 *Aeromonas* spp. isolates from seawater were 100% MDR and specifically showed complete resistance to b-lactamases and sulfonamides. In general, the presence of MDR bacteria is eventually due to various human activities, municipal or hospital waste stream, livestock enterprise runoff, effluents of pharmaceutical plants, or disposal of unused drugs that result in the spread of resistant bacteria, antibiotic residues, or both in the environment [48,50].

In this study, all tested strains were completely resistant to AM, P, and SXT (100%) and highly resistant to oxytetracycline (90%), with a considerable percentage of resistance (62.22%) to streptomycin. Furthermore, tested strains showed lower resistance (17-23%) to AK, NOR, C, CN, and NA (Table-3). The current finding is consistent with earlier reports and can be explained by the inherent resistance of *Aeromonas* spp. to beta-lactams and first-generation cephalosporins [51,52]. However, in a recent study on seawater samples collected from the South and North Coasts of Mediterranean Sea in Italy, less resistant isolates were detected with most of them exhibiting resistance to a single antibiotic, while only a single isolate showed resistance to two drugs and others were susceptible to all tested antibiotics [25].

Meanwhile, all *Aeromonas* isolates in the present study were susceptible to CTX, which was in line with other reports where *Aeromonas* are found typically sensitive to cephalosporins from the later generation [52,53]. This means that such compounds could be indicated in the treatment of confirmed *Aeromonas* diseases in the study area; however, with the increasing concerns about newly developed resistant pathogens, appropriate antibiotic usage, and alternate therapies for *Aeromonas* spp. infection should be implemented.

Although β-lactams are usually the antibiotics of choice for the treatment of bacterial infections, their efficacy has declined dramatically in the last decade due to the production of β-lactamases by resistant strains of bacteria. *bla*_TEM_ is the most frequently detected β-lactamase. Its expression results in not only penicillin resistance but also the development of TEM-type extended-spectrum β-lactamases due to various point mutations in *bla*_TEM_ gene, resulting in combined penicillins and broad-spectrum cephalosporin resistance [54]. Data in the present study confirmed the presence of *bla*_TEM_, *sul1*, and *tetA(A)* resistance genes in all XDR isolates. These findings were consistent with data available in the relevant literature, which confirmed the presence of bla_TEM_ in all *Aeromonas* isolates (100%) [55,56]. In another study in Egypt, *bla*_TEM_ gene from Nile tilapia and cat fish samples has been detected in all *A. hydrophila* studied strains (100%), while 83.33% of *A. caviae* carry the *bla*_TEM_ gene [57]. Interestingly, in an earlier study, *bla*_TEM_ gene was not detected from these *Aeromonas* strains from rainbow trout although there was β-lactam resistance phenotype [58].

Similarly, *sul1* resistance gene had been detected at a high rate in *Aeromonas* spp. from rainbow trout (87.1%) [58] and *Oreochromis niloticus* and *Clarias gariepinus* (75%) [57]. In another study, although *sul1* was present in *A. hydrophila* (41%, 7/17), it cannot be detected in *A. sobria* and *A. caviae* [59]. While variable occurrences of *tetA(A)* resistance gene had been reported in *Aeromonas* from several studies elsewhere, 50% [58], 87.5% [57], and 67.44% [60], in another Egyptian study, only *A. sobria* and *A. caviae* carry *tetA(A)* resistance gene, but *A. hydrophila* and *A. schubertii* did not carry it [56].

Although tested *Aeromonas* isolates were phenotypically resistant to streptomycin in the present study, the *aadA1* gene has not been detected in any of them. However, in other studies, *aadA1* gene has been detected at a higher rate of 70% [58] and 62.5% [57]. Such antibiotic resistance genes may end up in horizontal gene transfer between various bacterial species and strains, leading to multiple antibiotic resistances. This is especially a serious public health concern because it leads to treatment failure and weakens the effectiveness of drugs [48].

Enterotoxins represented in the cytotoxic gene (*act*) and two cytotonic genes (*alt* and *ast*) are declared to be crucial products implicated in human diarrheal disease [61]. As observed in the present study, all XDR *Aeromonas* carry the cytotoxic *act* gene (100%); however, *alt* gene was detected in only one *A. sobria* isolate (12.5%) (Table-4). This supports the findings in the previous studies where *alt* gene was confirmed in *A. sobria* but not in *A. caviae* [62]. Moreover, *ast* gene (6%) and *act* gene (63%) had been detected in *Aeromonas* spp. from various fish species and water in an earlier study [63]. However, *alt* gene has been detected in *A. hydrophila* from freshwater *M. cephalus* in a recent study in Egypt [42]. We failed to detect *ast* gene in any isolate in the current investigation. This is consistent with the finding of the most recent study in Vietnam [52]. However, in an earlier study in Egypt on channel catfish and tilapia, 70% of *A. hydrophila* isolates carry *ast* gene [57].

This wide variation in the prevalence of resistance and enterotoxin genes may be due to strain geographical dispersion, contamination levels in the surrounding effluents, and probably horizontal gene transfer [14]. Our findings add data to previous reports about MDR bacteria from the marine environment and suggest that despite the opportunistic and ubiquitous nature of *A. hydrophila* complex in fresh and marine water, fish, coastal water, and sewage; we may face a terrible ghost of a devastating impact if they are converted to be widely distributed MDR or XDR pathogens.

## Conclusion

Data in the present study highlight the role of environmental *Aeromonas* strains from wild mullet and seawater as reservoirs for antibiotic resistance and virulence genes that may pose a risk to human consumers either as food-borne infection or through contact with public health implications where efficacy of human treatment is in doubt. The high detection rate of *Aeromonas* spp. from the surface of the fish represents a potential occupational hazard to fish handlers through wound contamination in fishermen, aquaculture workers, housewives, and fishing fans along the coast of Port Said Governorate. The inadvertent use of antibiotics generally in all situations, including viral infections, is useless and promotes the development of bacterial resistance. The presence of such XDR and cytotoxic *Aeromonas* populations in fish and water poses a public health concern and emphasizes the urgent need for regular and continuous monitoring. Data presented in the current study may provide a baseline for further epidemiological and genetic studies that can better understand the origins of these resistant bacteria in the study area.

## Authors’ Contributions

HME and AMS: Designed the study. HME, AMS, HSE, NHE, HaME, and MMS: Collected the samples, performed the experiments, analyzed the data, and drafted and revised the manuscript. All the authors read and approved the final manuscript.
